# Adversarially Robust Learning *via* Entropic Regularization

**DOI:** 10.3389/frai.2021.780843

**Published:** 2022-01-04

**Authors:** Gauri Jagatap, Ameya Joshi, Animesh Basak Chowdhury, Siddharth Garg, Chinmay Hegde

**Affiliations:** Electrical and Computer Engineering, New York University, New York, NY, United States

**Keywords:** adversarial learning, robustness, adversarial attack, regularization, neural network training

## Abstract

In this paper we propose a new family of algorithms, ATENT, for training adversarially robust deep neural networks. We formulate a new loss function that is equipped with an additional entropic regularization. Our loss function considers the contribution of adversarial samples that are drawn from a specially designed distribution in the data space that assigns high probability to points with high loss and in the immediate neighborhood of training samples. Our proposed algorithms optimize this loss to seek adversarially robust valleys of the loss landscape. Our approach achieves competitive (or better) performance in terms of robust classification accuracy as compared to several state-of-the-art robust learning approaches on benchmark datasets such as MNIST and CIFAR-10.

## 1 Introduction

Deep neural networks have led to significant breakthroughs in the fields of computer vision (Krizhevsky et al., 2012), natural language processing (Zhang et al., 2020), speech processing (Carlini et al., 2016), recommendation systems (Tang et al., 2019) and forensic imaging (Rota et al. (2016)). However, deep networks have also been shown to be very susceptible to carefully designed “attacks” (Goodfellow et al., 2014; Papernot et al., 2016; Biggio and Roli, 2018). In particular, the outputs of networks trained via traditional approaches are rather brittle to maliciously crafted perturbations in both input data as well as network weights (Biggio et al., 2013).

Formally put, suppose the forward map between the inputs *x* and outputs *y* is modeled *via* a neural network as *y* = *f*(*w*; *x*) where *w* represents the set of trainable weight parameters. For a classification task, given a labeled dataset {*x*_*i*_, *y*_*i*_}, *i* = 1, … , *n* where *X* and *Y* represents all training data pairs, the standard procedure for training neural networks is to seek the weight parameters *w* that minimize the empirical risk:

$$\hat{w}=\arg\underset{w}{\min}\frac{1}{n}\overset{n}{\underset{i=1}{\sum }}L\left(f\left(w;x_{i}\right),y_{i}\right)≔\arg\underset{w}{\min}L\left(X;Y,w\right).$$

However, the prediction $\hat{y}\left(x\right)=f\left(\hat{w};x\right)$ can be very sensitive to changes in both $\hat{w}$ and *x*. For example, if a bounded perturbation to a test image input (or to the neural network weights) is permitted, i.e., ${\hat{y}}_{i}=f\left(\hat{w};x_{i}+\delta_{i}\right)$ where *δ*_*i*_ represents the perturbation, then the predicted label $\hat{y_{i}}$ can be made *arbitrarily* different from the true label *y*_*i*_.

Several techniques for finding such adversarial perturbations have been put forth. Typically, this can be achieved by maximizing the loss function within a neighborhood around the test point *x* (Tramèr et al., 2017; Madry et al., 2018):

$${\bar{x}}_{\text{worst}}=\arg\underset{\delta \in \Delta_{p}}{\max}L\left(f\left(\hat{w};x+\delta \right),y\right),$$ (1)

where $\hat{w}$ are the final weights of a pre-trained network. The perturbation set Δ_*p*_ is typically chosen to be an *ℓ*_*p*_-ball for some *p* ∈ {0, 1, 2, *∞*}.

The existence of adversarial attacks motivates the need for a “defense” mechanism that makes the network under consideration more robust. Despite a wealth of proposed defense techniques, the jury is still out on how optimal defenses should be constructed (Athalye et al., 2018).

We discuss several families of effective defenses. The first involves *adversarial training* (Madry et al., 2018). Here, a set of adversarial perturbations of the training data is constructed by solving a min-max objective of the form:

$$\hat{w}=\underset{w}{\min}\underset{\delta \in \Delta_{p}}{\max}\frac{1}{n}\overset{n}{\underset{i=1}{\sum }}L\left(f\left(w;x_{i}+\delta \right),y_{i}\right).$$

Wong and Kolter (2018) use a convex outer adversarial polytope as an upper bound for worst-case loss in robust training; here the network is trained by generating adversarial as well as few non-adversarial examples in the convex polytope of the attack via a linear program. Along the same vein include a mixed-integer programming based certified training for piece-wise linear neural networks (Tjeng et al., 2018) and integer bound propagation (Gowal et al., 2019).

The last family of approaches involves *randomized smoothing*. Here, both training the network as well as the inference made by the network are smoothed out over several stochastic perturbations of the target example (Lecuyer et al., 2019; Cohen et al., 2019; Salman et al., 2019a). This has the effect of optimizing a smoothed-adversarial version of the empirical risk. Randomized smoothing has also been used in combination with adversarial training (Salman et al., 2019b) for improved adversarial robustness under *ℓ*_2_ attacks^1^.

In this paper, we propose a new approach for training adversarially robust neural networks. The key conceptual ingredient underlying our approach is *entropic regularization*. Borrowing intuition from Chaudhari et al. (2019), instead of the empirical risk (or its adversarial counterpart), our algorithm instead optimizes over a local entropy-regularized version of the empirical risk:

$$\begin{array}{ll} \hat{w} & =\arg\underset{w}{\min}L_{DE},\\ L_{DE} & =\int_{X^{\prime }}L\left(X^{\prime };Y,w\right)\left[\frac{e^{\left(L\left(X^{\prime };Y,w\right)-\frac{\gamma }{2}\|X-X^{\prime }{\|}_{p}^{p}\right)}}{Z}\right]dX^{\prime }. \end{array}$$ (2)

Intuitively, this new loss function can be viewed as the convolution of the empirical risk with a Gibbs-like distribution to sample points from the neighborhoods, *X*′, of the training data points *X* that have high loss. Therefore, compared to adversarial training, we have replaced the inner maximization with an expected value with respect to a modified Gibbs measure which is matched to the geometry of the perturbation set.

**FIGURE 1:**
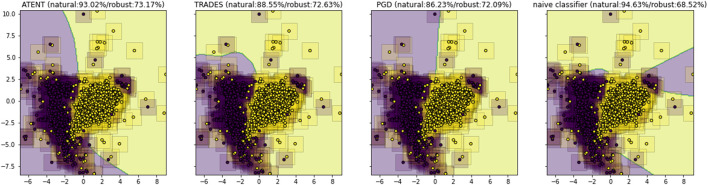
TSNE visualization of decision boundaries for a 3-layer neural network trained using different defenses; corresponding natural and robust test accuracies against *ℓ*_*∞*_ attacks for classifying MNIST digits 5 and 8.

Since the above loss function is difficult to optimize (or even evaluate exactly), we instead approximate it via Monte Carlo techniques. In particular, we use Stochastic Gradient Langevin Dynamics (Welling and Teh, 2011); in this manner, our approach blends in elements from adversarial training, randomized smoothing, and entropic regularization. We posit that the combination of these techniques will encourage a classifier to learn a better robust decision boundary as compared to prior art (see visualization in Figure 1).

To summarize, our specific contributions are as follows:

1. We propose a new entropy-regularized loss function for training deep neural networks (Eq. 2) that is a robust version of the empirical risk.
2. We propose a new Monte Carlo algorithm to optimize this new loss function that is based on Stochastic Gradient Langevin Dynamics. We call this approach Adversarial Training with ENTropy (ATENT).
3. We show that ATENT-trained networks provide improved (robust) test accuracy when compared to existing defense approaches.
4. We combine randomized smoothing with ATENT to show competitive performance with the smoothed version of TRADES.

In particular, we are able to train an *ℓ*_*∞*_-robust CIFAR-10 model to 57.23% accuracy at PGD attack level *ϵ* = 8/255, which is higher than the latest benchmark defenses based on both adversarial training using early stopping (Salman et al., 2019b) (56.8%) as well as TRADES (56.6%) (Zhang et al., 2019b).

## 2 Prior Work

Evidence for the existence of adversarial inputs for deep neural networks is by now well established (Carlini N. and Wagner D. A., 2017; Dathathri et al., 2017; Goodfellow et al., 2015; Goodfellow, 2018; Szegedy et al., 2013; Moosavi-Dezfooli et al., 2017). In image classification, the majority of attacks have focused on the setting where the adversary confounds the classifier by adding an imperceptible perturbation to a given input image. The range of the perturbation is pre-specified in terms of bounded pixel-space *ℓ*_*p*_-norm balls. Specifically, an *ℓ*_*p*_- attack model allows the adversary to search over the set of input perturbations Δ_*p*,*ϵ*_ = {*δ*: ‖*δ*‖_*p*_ ≤ *ϵ*} for *p* = {0, 1, 2, *∞*}.

Initial attack methods, including the Fast Gradient Sign Method (FGSM) and its variants (Goodfellow et al., 2014; Kurakin et al., 2016), proposed techniques for generating adversarial examples by ascending along the sign of the loss gradient:

$$x_{adv}=x+\epsilon \mathrm{sgn}\left(\nabla_{x}L\left(f\left(\hat{w};x\right),y\right)\right),$$

where (*x*_*adv*_ − *x*) ∈ Δ_*∞*,*ϵ*_. Madry et al. (2018) proposed a stronger adversarial attack via projected gradient descent (PGD) by iterating FGSM several times, such that

$$x^{t+1}=\Pi_{x+\Delta_{p,\epsilon }}\;{(x}^{t}+\alpha \mathrm{sgn}\left(\nabla_{x}L\left(f\left(\hat{w};x\right),y\right)\right),$$

where *p* = {2, *∞*}. These attacks are (arguably) the most successful available attack techniques reported to date, and serve as the starting point for our comparisons. Both Deep Fool Moosavi-Dezfooli et al., 2016) and Carlini-Wagner (Carlini N. and Wagner D., 2017) construct an attack by finding smallest possible perturbation that can flip the label of the network output.

Several strategies for defending against attacks have been developed. In Madry et al. (2018), adversarial training is performed via the min-max formulation Eq. 1. The inner maximization is solved using PGD, while the outer objective is minimized using stochastic gradient descent (SGD) with respect to *w*. This can be slow to implement, and speed-ups have been proposed in Shafahi et al. (2019) and Wong et al. (2020). In Li B. et al. (2018); Cohen et al. (2019); Lecuyer et al. (2019); Salman et al. (2019a, Salman et al. (2019b), the authors developed certified defense strategies via randomized smoothing. This approach consists of two stages: the first stage consists of training with noisy samples, and the second stage produces an ensemble-based inference. See Ren et al. (2020) for a more thorough review of the literature on various attack and defense models.

Apart from minimizing the worst case loss, approaches which minimize the upper bound on worst case loss inclu Wong et al., 2018; Tjeng et al. (2018); Gowal et al. (2019). Another breed of approaches use a modified loss function which considers surrogate adversarial loss as an added regularization, where the surrogate is cross entropy (Zhang et al., 2019b) (TRADES), maximum margin cross entropy (Ding et al., 2019) (MMA) and KL divergence (Wang et al., 2019) (MART) between adversarial sample predictions and natural sample predictions.

In a different line of work, there have been efforts towards building neural network networks with improved generalization properties. In particular, heuristic experiments by Hochreiter and Schmidhuber (1997); Keskar et al. (2016); Li H. et al. (2018) suggest that the loss surface at the final learned weights for well-generalizing models is relatively “flat”^2^. Building on this intuition, Chaudhari et al. (2019) showed that by explicitly introducing a smoothing term (via entropic regularization) to the training objective, the learning procedure weights towards regions with flatter minima by design. Their approach, Entropy-SGD (or ESGD), is shown to induce better *generalization* properties in deep networks. We leverage this intuition, but develop a new algorithm for training deep networks with better *adversarial robustness* properties. We also highlight some papers written concurrently in Supplementary Material.

## 3 Problem Formulation

The task of classification, given a training labelled dataset $\left\{x_{i}\in X,y_{i}\right\}$ , *i* ∈ {1, … , *n*}, consists of solving the standard objective by optimizing weight parameters *w*, $\min_{w}\frac{1}{n}\sum_{i=1}^{n}L\left(f\left(w;x_{i}\right),y_{i}\right)$ where *y*_*i*_ is a one-hot class encoding vector of length *m* and *m* is the total number of classes. The training data matrix itself is represented using shorthand $X\in R^{n\times d}$ and labels in $Y\in R^{n}$ where we have access to *n* training samples which are *d*-dimensional each. Given this formulation, the primary task is to minimize the cross-entropy Loss function $L\left(w;X,Y\right)=-\frac{1}{n}\sum_{i=1}^{n}\sum_{j=1}^{m}y_{i,j}\log{\hat{y}}_{i,j}$ . In this paper, we design an augmented version of the loss function L which models a class of adversarial perturbations and also introduce a new procedure to minimize it.

We first recap the Entropy SGD (Chaudhari et al., 2019) (see also Supplementary Material). Entropy-SGD considers an augmented loss function of the form

$$L_{ent}\left(w;X,Y\right)=-\log\int_{w^{\prime }}e^{-L\left(w^{\prime };X,Y\right)-\frac{\gamma }{2}\|w-w^{\prime }{\|}_{2}^{2}}dw^{\prime }.$$

By design, minimization of this augmented loss function promotes minima with wide valleys. Such a minimum would be robust to perturbations in *w*, but is not necessarily advantageous against adversarial data samples *x*_*adv*_. In our experiments (Section 4) we show that networks trained with Entropy-SGD perform only marginally better against adversarial attacks as compared to those trained with standard SGD.

For the task of adversarial robustness, we instead develop a data-space version of Entropy-SGD. To model for perturbations in the samples, we design an augmented loss that regularizes the data space. Note that we only seek specific perturbations of data *x* that *increase* the overall loss value of prediction. In order to formally motivate our approach, we first make some assumptions.

Assumption 1 The distribution of possible adversarial data inputs of the neural network obeys a positive exponential distribution of the form below, where the domain of L(X;Y,w) is bounded:

$$p\left(X;Y,w,\beta \right)=\left\{\begin{array}{cc} Z_{w,\beta }^{-1}e^{\beta L\left(X;Y,w\right)}\; & \text{if }\;L\left(X;Y,w\right)\leq R,\\ 0\; & \text{if }\;L\left(X;Y,w\right)>R, \end{array}\right.$$ (3)

and Zw,β is the partition function that normalizes the probability distribution.

Note here that cross entropy loss L is always lower bounded as L≥0 . flushleft

Intuitively, the neural network is more likely to “see” perturbed examples from the adversary corresponding to higher loss values as compared to lower loss values. The parameter *R* is chosen to ensure that the integral of the probability curve is bounded. When the temperature parameter *β* → ∞, the above Gibbs distribution concentrates at the maximizer(s) of $L\left(\bar{X};Y,w\right)$ , where $\bar{X}$ is the “worst possible” set of adversarial inputs to the domain of the loss function for fixed weights *w*. For a given attack ball Δ_*p*,_ϵ with radius ϵ and norm *p*, and fixed weights *w*, this value equates to:

$$\bar{X}=\arg\underset{X^{\prime }\in \Delta_{p,\epsilon }}{\max}L\left(X^{\prime };X,Y,w\right),$$

where $\max_{X^{\prime }\in \Delta_{p,\epsilon }}L\left(X^{\prime };X,Y,w\right)\leq R$ .

Assumption 2 A modified distribution, (without loss of generality, setting *β* = 1) with an additional smoothing parameter, assumes the form:

$$p\left(X^{\prime };X,Y,w,\gamma \right)=\left\{\begin{array}{cc} Z_{X,w,\gamma }^{-1}e^{L\left(X^{\prime };Y,w\right)-\frac{\gamma }{2}\|X^{\prime }-X{\|}_{F}^{2}}\; & \text{if}\;L\left(X^{\prime };Y,w\right)\leq R\\ 0\; & \text{if}\;L\left(X^{\prime };Y,w\right)>R \end{array}\right.$$

where Z_X,w,γ_ is the partition function that normalizes the probability distribution.

Here *γ* controls the penalty of the distance of the adversary from true data *X*; if *γ* → *∞*, the sampling is sharp, i.e. *p*(*X*′ = *X*; *X*, *Y*, *w*, *γ*) = 1 and *p*(*X*′ ≠ *X*; *X*, *Y*, *w*, *γ*) = 0, which is the same as sampling only the standard loss L , meanwhile *γ* → 0 corresponds to a uniform contribution from all possible data points in the loss manifold.

**FIGURE 2:**
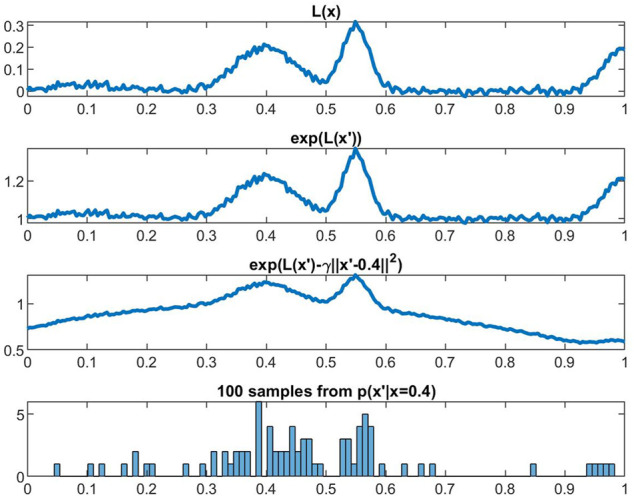
Illustration of the sampling procedure in Assumption 2 at fixed weights *w*. The distribution produces samples *x*′ from distribution *p*(*x*′|*x* = 0.4), and we compute the average loss over these samples. Effectively, this encourages ATENT to search for *w* where *L*(*x*; *w*) is relatively flat in the neighborhood of *x*.

Now, we develop an augmented loss function which incorporates the probabilistic formulation in Assumption 2. The standard objective can be re-written as the functional convolution:

$$\underset{w}{\min}L\left(w;X,Y\right)≔\underset{w}{\min}\int_{X^{\prime }}L\left(X^{\prime };Y,w\right)\delta \left(X-X^{\prime }\right)dX^{\prime },$$

which can be seen as a sharp sampling of the loss function at training points *X*. Now, define the *Data-Entropy Loss*:

$$L_{DE}\left(w;X,Y,\gamma \right)=\int_{X^{\prime }}L\left(X^{\prime };Y,w\right)p\left(X^{\prime };X,Y,w,\gamma \right)dX^{\prime }$$ (4)

our new objective is to minimize this augmented objective function $L_{DE}\left(w;X,Y,\gamma \right)$ , which resembles expected value of the standard loss function sampled according to a distribution that (i) penalizes points further away from the true training data 2) boosts data points which correspond to high loss values. Specifically, the adversarial samples generated by the distribution in Assumption 2 will correspond to those with *high loss* values in the *immediate neighborhood* of the true data samples. This sampling process is also described in Figure 2. We also highlight theoretical properties of our augmented loss function *via* Lemma 3.1, proof of which can be found in of supplement B.

Lemma 3.1 The effective loss $F\left(X^{\prime };X,Y,w\right)≔\frac{\gamma }{2}\|X-X^{\prime }{\|}_{F}^{2}-L\left(X^{\prime };Y,w\right)$ which guides the Langevin sampling process in Eq. 8 is

 1. *β* + γ smooth if L(X;Y,w) is β-smooth in X*.*
2. $\left(\frac{\gamma }{4},\frac{L^{2}}{\gamma }+\frac{\gamma }{2}\|X{\|}_{F}^{2}\right)$ dissipative if L(X;Y,w) is L-Lipschitz in X.

If gradient descent is used to minimize the loss in Eq. 4, the gradient update corresponding to the augmented loss function can be computed as follows

$$\begin{array}{ll} \nabla_{w}L_{de}\left(w;X,Y,\gamma \right) & =\nabla_{w}\int_{X^{\prime }}L\left(X^{\prime };Y,w\right)p\left(X^{\prime };X,Y,w,\gamma \right)dX^{\prime }\\ & =\nabla_{w}E_{X^{\prime }∼p\left(X^{\prime };X,Y,w,\gamma \right)}\left[L\left(X^{\prime };Y,w\right)\right] \end{array}$$ (5)

Correspondingly, the weights of the network, when trained using gradient descent, using Eq. 5, can be updated as

$$w^{+}=w-\eta \nabla_{w}E_{X^{\prime }∼p\left(X^{\prime };X,Y,w,\gamma \right)}\left[L\left(X^{\prime };Y,w\right)\right]$$ (6)

where *η* is the step size. The expectation in Eq. 5 is carried out over the probability distribution of data samples *X′* as defined in Assumption 2. This can be seen as the adversarial version of the formulation developed in Entropy SGD (Chaudhari et al. (2019)), where the authors use a Gibbs distribution to model an augmented loss function that explores the loss surface at points that are perturbed from the current weights *w*, denoted by *w*′ (see Supplementary Material). In contrast, in our approach, we consider loss contributions from perturbations *X*′ of data points *X*. This analogue is driven by the fact that the core objective in Chaudhari et al. (2019) is to design a network which is robust to perturbations in *weights* (generalization), where as the core objective of this paper is to design a network that is robust to perturbations in the *inputs* (adversarial robustness).

The expectation in Eq. 5 is computationally intractable to optimize (or evaluate). However, using the Euler discretization of the Langevin Stochastic Differential Equation (Welling and Teh, 2011), it can be approximated well. Samples can be generated from *p*(*X*′) as:

$$X^{\prime k+1}=X^{\prime k}+\eta^{\prime }\nabla_{X^{\prime }}\log p\left(X^{\prime t}\right)+\sqrt{2\eta^{\prime }}\varepsilon N\left(0,I\right)$$ (7)

where *η*′ is the step size for Langevin sampling, *ɛ* is a scaling factor that controls additive noise. In Langevin dynamics, when one considers a starting point of *X*′^0^ then the procedure above yields samples *X*′^1^ … *X*′^*t*^ that follow the distribution *p*(*X*′). Intuitively, the stochastic process *X*′^*t*^ is more likely to visit points in the immediate neighborhoods of the *entire training* dataset *X* corresponding to high loss values.

Observe that *X*′ and *X* have the same dimensions and the gradient term in the above equation needs to be computed over *n*, *d*-dimensional data points. In practice this can be computationally expensive. Therefore, we discuss a stochastic variant of this update rule, which considers mini-batches of training data instead. Plugging in the distribution in Eq.3, and using the Euler discretization for Langevin Stochastic Differential Equations, the update rule for sampling *X*′ is

$$\begin{array}{ll} \frac{X^{\prime k+1}-X^{\prime k}}{\eta^{\prime }} & =\nabla_{X^{\prime k}}\left(L\left(X^{\prime k};Y,w\right)-\frac{\gamma }{2}\|X-X^{\prime k}{\|}_{F}^{2}\right)+\sqrt{\frac{2\varepsilon^{2}}{\eta^{\prime }}}N\left(0,I\right)\\ & =\nabla_{X^{\prime k}}L\left(X^{\prime k};Y,w\right)+\gamma \left(X-X^{\prime k}\right)+\sqrt{\frac{2\varepsilon^{2}}{\eta^{\prime }}}N\left(0,I\right) \end{array}$$ (8)

where we have incorporated *Z*_*X*,*wγ*_ in the step size *η*′. Note that as the number of updates *k* → *∞*, the estimates from the procedure in Eq. 8 converge to samples from the true distribution. *p*(*X* ^′^; *X*, *Y*, *w*, *γ*). We then want to estimate $\nabla_{w}L_{de}\left(w;X,Y,\gamma \right)=\nabla_{w}E_{X^{\prime }∼p\left(X^{\prime }\right)}\left[L\left(w;X^{\prime },Y,\gamma \right)\right]$ using the samples obtained from the above iterative procedure. Chaudhari et al. (2019), use an exponentially decaying averaging process to estimate the expected value.

***Batch-wise updates for stochastic gradient estimates:*** As is typical with large datasets, instead of using the entire training data for computing gradients in Eq. 5 and Eq. 7, one can use batch-wise data where the training data is segmented into *J* batches $\left[X_{B_{1}},X_{B_{2}}\ldots X_{B_{J}}\right]$ . This is essentially a combination of Stochastic Gradient Descent and Langevin Dynamics and is known as Stochastic Gradient Langevin Dynamics in recent literature Welling and Teh, 2011).

This discussion effectively leads to the algorithm shown in Algorithm 1, which we refer to as Adversarial Training using Entropy (or ATENT), designed for *ℓ*_2_ attacks. Note that we have considered exponentially decaying averaging over sample loss *μ* ^*k*^ in Line 10 of Algorithm 1.

Algorithm 1 *ℓ*_2_-ATENT

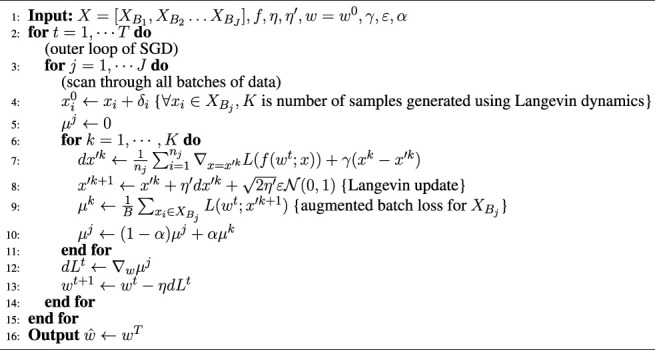

***Comparison to PGD Adversarial Training:*** (see also Algorithm 2 Madry et al., 2018 in Supplementary Material, referred as PGD-AT). It is easy to see that the updates of PGD-AT are similar to that of Algorithm 1, consisting broadly of two types of gradient operations in an alternating fashion—1) an (inner) gradient with respect to samples *X* (or batch-wise samples $X_{B_{j}}$ ) and 2) an (outer) gradient with respect to weights *w*. While PGD-AT minimizes the *worst-case* loss in an *ϵ*-neighborhood (specifically *ℓ*_2_ or *ℓ*_*∞*_ ball) of *X*, ATENT minimizes an *average loss* over our specifically designed probability distribution (Assumption 3) in the neighborhood of *X*. Note that the gradient operation in Eq. 8 is also the gradient for the regularized version of inner maximation of the adversarial training problem (Madry et al., 2018), but with added noise term,

$$\begin{array}{l} \underset{X^{\prime }}{\max}L\left(X^{\prime };X,Y,w\right)\;s.t.\;\|X^{\prime }-X{\|}_{F}^{2}\leq \epsilon \;\Leftrightarrow \;\underset{X^{\prime }}{\max}L\left(X^{\prime };X,Y,w\right)-\frac{\gamma }{2}\|X^{\prime }-X{\|}_{F}^{2} \end{array}$$ (9)

constraint being satisfied if ‖*X*′ − *X*‖_*F*_ is minimized, or − ‖*X*′ − *X*‖_*F*_ is maximized).

The width of the Gaussian smoothing is adjusted with *γ*, which is analogous to controlling the projection radius *ϵ* in the inner-maximization of PGD-AT. Then the second and third terms in Eq. 8 are simply gradient of an *ℓ*_2_-regularization term over data space *X*′ and noise. In this way, ATENT can be re-interpreted as a stochastic formalization of *ℓ*_2_-PGD-AT, with noisy controlled updates.

***Comparison to randomized smoothing:*** Cohen et al. (2019), describe a defense to adversarial perturbations, in the form of smoothing. A smoothed classifier *g*, under isotropic Gaussian noise $\varepsilon =N\left(0,\sigma^{2}I\right)$ , produces an output:

$$g\left(x\right)=\arg\underset{j}{\max}P\left(f\left(x+\varepsilon \right)=\;j\right).$$ (10)

where P denotes probability distribution (see Supplementary Material for detailed discussion). SmoothAdv Salman et al., 2019b) is an adversarial attack as well as defense for smoothed classifiers, which replaces standard loss with cross entropy loss of a smoothed classier. In comparison, we compute a smoothed version of the cross entropy loss of a standard classifier. This is similar to the setup of Blum et al. (2020) (TRADES with smoothing). The procedure in Algorithm 1 is therefore amenable to randomized smoothing in its evaluation. We discuss a smoothed evaluation of ATENT in the next section.

Algorithm 2 *ℓ*_*∞*_-ATENT

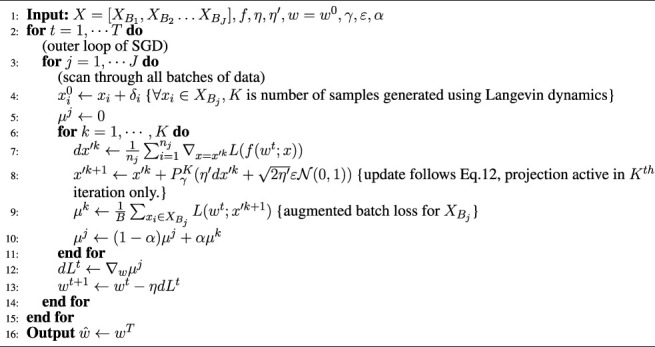

***Extension to defense against ℓ***_***∞***_ ***-attacks:*** It is evident that due to the isotropic structure of the Gibbs measure around each data point, Algorithm 1, *ℓ*_2_-ATENT is best suited for *ℓ*_2_ attacks. However this may not necessarily translate to robustness against *ℓ*_*∞*_ attacks. For this case, one can use an alternate assumption on the distribution of potential adversarial examples. flushleft

Assumption 3 We consider a modification of the distribution in Assumption 2 to account for robustness against ℓ_∞_ type attacks:

$$p\left(X^{\prime };X,Y,w,\gamma \right)=\left\{\begin{array}{cc} Z_{X,w,\gamma }^{-1}e^{\left(L\left(X^{\prime };Y,w\right)-\frac{\gamma }{2}\|X^{\prime }-X{\|}_{\infty }\right)}\; & \text{if}\;L\left(X^{\prime };Y,w\right)\leq R\\ 0\; & \text{if}\;L\left(X^{\prime };Y,w\right)>R \end{array}\right.$$

*where* ‖⋅‖_*∞*_ *is the ℓ*_*∞*_ *norm on the vectorization of its argument and Z*_*X*,*w*,*γ*_ *normalizes the probability.*

The corresponding Data Entropy Loss for *ℓ*_*∞*_ defenses is: flushleft

$$L_{DE,\infty }\left(w;X,Y\right)=Z_{X,w,\gamma }^{-1}\int_{X^{\prime }}L\left(X^{\prime };Y,w\right)e^{\left(L\left(X^{\prime };Y,w\right)-\frac{\gamma }{2}\|X-X^{\prime }{\|}_{\infty }\right)}dX^{\prime }$$

This resembles a smoothed version of the loss function with a exponential *ℓ*_*∞*_ kernel along the data dimension to model points in the *ℓ*_*∞*_ neighborhood of *X* which have high loss. The SGD update to minimize this loss becomes:

$$\begin{array}{ll} \nabla_{w}L_{DE,\infty }\left(w;X,Y\right) & =\nabla_{w}E_{X^{\prime }∼p\left(X^{\prime }\right)}\left[L\left(w;X^{\prime },Y\right)\right]\\ \;\Rightarrow \;w^{+} & =w-\eta \nabla_{w}L_{DE,\infty }\left(w;X,Y\right) \end{array}$$

where the expectation over *p*(*X*′) is computed by using samples generated via Langevin Dynamics:

$$X^{\prime k+1}=X^{\prime k}+\eta^{\prime }\nabla_{X^{\prime }}\log p\left(X^{\prime k}\right)+\sqrt{2\eta^{\prime }}\varepsilon N\left(0,I\right)$$

Plugging in the distribution in Assumption 3 the update rule for sampling *X*′:

$$\begin{array}{ll} \frac{X^{\prime k+1}-X^{\prime k}}{\eta^{\prime }} & =\nabla_{X^{\prime k}}\left(L\left(X^{\prime k};Y,w\right)-\frac{\gamma }{2}\|X-X^{\prime k}{\|}_{\infty }\right)+\sqrt{\frac{2\varepsilon^{2}}{\eta^{\prime }}}N\left(0,I\right)\\ & =\nabla_{X^{\prime k}}L\left(X^{\prime k};Y,w\right)+\gamma \mathrm{sign}\left(X_{i}-X_{i}^{\prime k}\right)\cdot 1+\sqrt{\frac{2\varepsilon^{2}}{\eta^{\prime }}}N\left(0,I\right) \end{array}$$ (11)

where $i=\arg\max_{j}|X_{j}-X_{j}^{\prime k}|$ and *j* scans all elements of the tensors *X*, *X*′^*k*^ and **1**_*j*_ = *δ*_*i*,*j*_. The second term in the update rule navigates the updates *X*′^*k*+1^ to lie in the immediate *ℓ*_*∞*_ neighborhood of *X*. Note that this training process requires taking gradients of *ℓ*_*∞*_ distance. In the update rule in Eq. 11, the gradient update only happens along one coordinate. In practice when we test this update rule, the algorithm fails to converge. This is due to the fact that typically a sizeable number of elements of *X*′ − *X* have a large magnitude.

The expression in the penultimate step of Eq. 11, is the gradient of a regularized maximization problem,

$$\begin{array}{l} \underset{X^{\prime }}{\max}L\left(X^{\prime };X,Y,w\right)\\ s.t.\;\|X^{\prime }-X{\|}_{\infty }\leq \epsilon \;\Leftrightarrow \;\underset{X^{\prime }}{\max}L\left(X^{\prime };X,Y,w\right)-\gamma \|X^{\prime }-X{\|}_{\infty } \end{array}$$

where *γ* is inversely proportional to *ϵ* (constraint is satisfied if ‖*X*′ − *X*‖_*∞*_ is minimized, or − ‖*X*′ − *X*‖_*∞*_ is maximized). This expression can be maximized only if *X*′ ∈ Δ_*∞*,*ϵ*_ of *X*; however when we take gradients along only one coordinate, this may not be sufficient to drive all coordinates of *X*′ towards Δ_*∞*,*ϵ*_ of *X*.

Similar to the *ℓ*_*∞*_ Carlini Wagner attack (Carlini N. and Wagner D., 2017), we replace the gradient update of the *ℓ*_*∞*_ term, with a clipping based projection oracle. We design an accelerated version of the update rule in Eq. 11, in which we perform a clipping operation, i.e., an *ℓ*_*∞*_ ball projection of the form:

$$\begin{array}{ll} X^{\prime k+1}-X^{\prime k} & =\eta^{\prime }\nabla_{X^{\prime }}L\left(X^{\prime k};Y,w\right)+\sqrt{2\eta^{\prime }}\varepsilon N\left(0,I\right),\\ X^{\prime K}-X^{\prime K-1} & =P\gamma \left(\eta^{\prime }\nabla_{X^{\prime }}L\left(X^{\prime K-1};Y,w\right)+\sqrt{2\eta^{\prime }}\varepsilon N\left(0,I\right)\right) \end{array}$$ (12)

where element-wise projection *P*_*γ*_(*z*) = *z* if |*z*| < 1/*γ* and *P*_*γ*_(*z*) = 1/*γ* if |*z*| > 1/*γ*. Empirically, we also explored an alternate implementation where the projection takes place in each inner iteration *k*, however, we find the version in Algorithm 2 to give better results.

In both Algorithms 1 and 2, we initialize the Langevin update step with a random normal perturbation *δ*_*i*_ of benign samples, which is constructed to lie inside within approximately 1/*γ* radius of the natural samples.

## 4 Experiments

In this section we perform experiments on a five-layer convolutional model with 3 CNN and 2 fully connected layers, used in Zhang et al. (2019b); Carlini N. and Wagner D. (2017), trained on MNIST. We also train a WideResNet-34-10 on CIFAR10 [as used in Zhang et al. (2019b)] as well as ResNet20. Due to space constraints, we present supplemental results in Supplementary Material. We conduct our experiments separately on networks specifically trained for *ℓ*_2_ attacks and those trained for *ℓ*_*∞*_ attacks. We also test randomized smoothing for our *ℓ*_2_-ATENT model. Source code is provided in the supplementary material.

***Attacks:*** For *ℓ*_2_ attacks, we test PGD-40 with 10 random restarts, and CW2 attacks at radius *ϵ*_2_ = 2 for MNIST and PGD-40 and CW2 attacks at *ϵ*_2_ = 0.43 ( $\approx \epsilon_{\infty }=2/255$ ) and *ϵ*_2_ = 0.5 = 128/255 for CIFAR10. For *ℓ*_*∞*_ attacks, we test PGD-20, *ℓ*_*∞*_CW, DeepFool attacks at radiii *ϵ*_*∞*_ = 0.3 for MNIST and *ϵ*_*∞*_ = 0.031 = 8/255 for CIFAR10. We test ATENT at other attack radii in Supplementary Material. For implementing the attacks, we use the Foolbox library (Rauber et al., 2017) and the Adversarial Robustness Toolbox (Nicolae et al. (2018)).

***Defenses:*** We compare models trained using: SGD (vanilla), Entropy SGD (Chaudhari et al., 2019), PGD-AT (Madry et al., 2018) with random starts [or PGD-AT(E) with random start, early stopping (Rice et al., 2020)], TRADES (Zhang et al., 2019b), MMA (Ding et al., 2019) and MART (Wang et al., 2019). Wherever available, we use pretrained models to tabulate robust accuracy results for PGD-AT, TRADES, MMA and MART as presented in their published versions. Classifiers giving the best and second best accuracies are highlighted in each category. We note here that a good defense mechanism should give better robust accuracies across different attack strategies. Since no single defense strategy outperforms every other defenses across all attacks, we highlight the *best two* robust accuracies. We find that ATENT obtains either the best or second best robust accuracy against all methods tested. This suggests that ATENT generalizes better than other defense strategies against various attacks.

***Smoothing:*** We also test randomized smoothing (Cohen et al., 2019) in addition to our adversarial training to evaluate certified robust accuracies.

The results were generated using an Intel(R) Xeon(R) W-2195 CPU 2.30 GHz Lambda cluster with 18 cores and a NVIDIA TITAN GPU running PyTorch version 1.4.0.

### 4.1 MNIST

In Tables 1 and 2, we tabulate the robust accuracy for 5-layer convolutional network trained using the various approaches discussed above for both *ℓ*_2_ and *ℓ*_*∞*_ attacks respectively.

**TABLE 1:** Robust percentage accuracies of 5-layer convolutional net for MNIST against *ℓ*_2_, *ϵ* = 2 attack.

**Attack →**	**Benign Acc**	*ℓ*_2_ **PGD-40**	*ℓ*_2_ **CW**
*↓* **Defense**			
SGD	**99.38**	19.40	13.20
Entropy SGD	99.24	19.12	14.52
*ℓ*_2_ PGD-AT	98.76	72.94	-
TRADES	97.54	**76.08**	-
MMA	**99.27**	73.02	72.72
*ℓ*_2_ ATENT	98.66	**77.21**	**76.72**

The highest two accuracy values in each column are highlighted in bold.

**TABLE 2:** Robust accuracies (in percentages) of 5-layer convolutional net for MNIST against *ℓ*_*∞*_, *ϵ* = 0.3 attack.

**Attack →**	**Benign**	*ℓ*_*∞*_ ** PGD-20**	*ℓ*_*∞*_ **CW**
*↓* ** Defense**	**Acc**	*ϵ*_***∞***_ ** = 0.3**	*ϵ*_***∞***_ **= 0.3**
SGD	99.39	0.97	32.37
Entropy SGD	99.24	1.17	34.34
*ℓ*_*∞*_ PGD-AT	99.36	96.01	94.25
TRADES	**99.48**	96.07	94.03
MMA	98.92	95.25	94.77
MART	98.74	**96.48**	**96.10**
*ℓ*_*∞*_ ATENT	**99.45**	**96.44**	**97.40**

The highest two accuracy values in each column are highlighted in bold.

***Training setup:*** Complete details are provided in Supplementary Material. Our experiments for *ℓ*_2_ attack are presented in Table 1. We perform these experiments on a LeNet5 model imported from the Advertorch toolbox (architecture details are provided in the supplement). For *ℓ*_2_-ATENT we use a batch-size of 50 and SGD with learning rate of *η* = 0.001 for updating weights. We set *γ* = 0.05 and noise ε∼0.001N(0,I) . We perform *K* = 40 Langevin epochs and set the Langevin parameter *α* = 0.9, and step *η*′ = 0.25. For attack, we do a 40-step PGD attack with *ℓ*_2_-ball radius of *ϵ* = 2. The step size for the PGD attack is 0.25, consistent with the configuration in Ding et al. (2019). We perform early stopping by tracking robust accuracies of validation set and report the best accuracy found.

In Table 2, we use a SmallCNN configuration as described in Zhang et al. (2019b) (architecture in supplement). We use a batch-size of 128, SGD optimizer with learning rate of *η* = 0.01 for updating weights. We set *γ* = 3.33 and noise ε∼0.001N(0,I) . We perform *L* = 40 Langevin epochs and we set the Langevin parameter *α* = 0.9, and step *η*′ = 0.01, consistent with the configuration in Zhang et al. (2019b). For the PGD attack, we use a 20-step PGD attack with step-size 0.01, for *ℓ*_*∞*_-ball radius of *ϵ* = 0.3. We perform an early stopping by tracking robust accuracies on the validation set and report the best accuracy found. Other attack configurations can be found in the supplement.

Our experiments on the Entropy-SGD (row 2 in Tables 1 and 2) trained network suggests that networks trained to find flat minima (with respect to weights) are not more robust to adversarial samples as compared to vanilla SGD.

### 4.2 CIFAR10

Next, we extend our experiments to CIFAR-10 using a WideResNet 34-10 as described in Zhang et al. (2019b); Wang et al. (2019) as well as ResNet-20. For PGD-AT (and PGD-AT (E)), TRADES, and MART, we use the default values stated in their corresponding papers.

***Training setup:*** Complete details in Supplementary Material. Robust accuracies of WRN-34-10 classifer trained using state of art defense models are evaluated at the *ℓ*_*∞*_ attack benchmark requirement of radius *ϵ* = 8/255, on CIFAR10 dataset and tabulated in Table 3. For *ℓ*_*∞*_-ATENT, we use a batch-size of 128, SGD optimizer for weights, with learning rate *η* = 0.1 (decayed to 0.01 at epoch 76), 76 total epochs, weight decay of 5 × 10^–4^ and momentum 0.9. We set *γ* = 1/(0.0031), *K* = 10 Langevin iterations, ε=0.001N(0,I) , at step size *η*′ = 0.007. We test against 20-step PGD attack, with step size 0.003, as well as *ℓ*_*∞*_-CW and Deep Fool attacks using FoolBox. *ℓ*_*∞*_-ATENT is consistently among the top two performers at benchmark configurations.

**TABLE 3:** Robust accuracies of WRN34-10 net for CIFAR10 against *ℓ*_*∞*_ attack of *ϵ* = 8/255.

**Defense →**	**PGD**	**TRADES**	**MART**	**ATENT**
*↓* ** Attack**	**AT**			***ℓ***_***∞***_
Benign	**87.30**	84.92	84.17	**85.67**
*ℓ*_*∞*_ PGD-20	47.04	56.61	**57.39**	**57.23**
(E)	56.80			
*ℓ*_*∞*_ CW	49.27	**62.67**	54.53	**62.34**
*ℓ*_*∞*_ DeepFool	-	**58.15**	55.89	**57.21**

The highest two accuracy values in each row are highlighted.

***Importance of early stopping:*** Because WRN34-10 is highly overparameterized with approximately 48 million trainable parameters, it tends to overfit adversarially-perturbed CIFAR10 examples. The success of TRADES (and also PGD) in Rice et al. (2020) relies on an early stopping condition and corresponding learning rate scheduler. We strategically search different early stopping points and report the best possible robust accuracy from different stopping points.

We test efficiency of our *ℓ*_2_-based defense on both *ℓ*_2_ attacks, as well as compute *ℓ*_2_ certified robustness for the smoothed version of ATENT against smoothed TRADES (Blum et al., 2020) in Supplementary Table S2 in Supplementary Material. We find that our formulation of *ℓ*_2_ ATENT is both robust against *ℓ*_2_ attacks, as well as gives a competitive certificate against adversarial perturbations for ResNet20 on CIFAR10.

In Supplementary Material we also demonstrate a fine-tuning approach for ATENT, where we consider a pre-trained WRN34-10 and fine tune it using ATENT, similar to the approach in Jeddi et al. (2020). We find that ATENT can be used to fine tune a naturally pretrained model at lower computational complexity to give competitive robust accuracies while almost retaining the performance on benign data.

### 4.3 Discussion

We propose a new algorithm for defending neural networks against adversarial attacks. We demonstrate competitive (and often improved) performance of our family of algorithms (ATENT) against the state of the art. We analyze the connections of ATENT with both PGD-adversarial training as well as randomized smoothing. Future work includes extending to larger datasets such as ImageNet, as well as theoretical analysis for algorithm convergence.

## Data Availability

The original contributions presented in the study are included in the article/Supplementary Material, further inquiries can be directed to the corresponding authors.

## Footnotes

1 This family of methods has the additional benefit of being certifiably robust: all points within a ball of a given radius around the test point are provably classified with the correct label.

2 This is not strictly necessary, as demonstrated by good generalization at certain sharp minima (Dinh et al., 2017).
