# Supplementary material for: Adversarially Robust Learning via Entropic Regularization
Source: Front Artif Intell. 2022 Jan 4;4:780843. doi: 10.3389/frai.2021.780843 (PMC8764444; doi:10.3389/frai.2021.780843)
Supplement: Supplementary file 1 [file Presentation1.pdf]

Supplementary Material

APPENDICES

A ADDITIONAL EXPERIMENTS AND DETAILS

In this section, we provide additional details as well as experiments to supplement those in Section 4.

Table S1. Percentage robust accuracies of ResNet-20 for CIFAR10 against ℓ_2 attack.

$\downarrow$ Algorithm/Attack$\rightarrow$	Model	Training param	Benign	PGD-10 ϵ_2 = 0.5	PGD-10 ϵ_2 = 1
PGD-AT	WideResNet28-4	$\epsilon_2 = 1$	83.25	66.69	46.11
MMA	WideResNet28-4	$d = 1$	**88.92**	**66.81**	37.22
ℓ_2 ATENT	ResNet20	$\gamma = 0.05$, $\varepsilon = 0.001\mathcal{N}(0, \mathbf{1})$	85.44	65.12	**47.38**
					ϵ_2=0.435
TRADES (smooth)	ResNet20	$\epsilon_2 = 0.435$, $\sigma = 0.12$	**75.13**		61.03
ℓ_2 ATENT	ResNet20	$\gamma = 0.05$, $\sqrt{2\eta'}\varepsilon = 0.12\mathcal{N}(0, \mathbf{1})$	72.10		**64.53**

A.1 Detailed training setup

Architectures: For MNIST- ℓ_∞ experiments, we consider a CNN architecture with the following configuration (same as Zhang et al. (2019b)). Feature extraction consists of the following sequence of operations: two layers of 2-D convolutions with 32 channels, kernal size 3, ReLU activation each, followed by maxpooling by factor 2, followed by two layers of 2-D convolutions with 64 channels, kernel size 3, ReLU activation, and finally another maxpool (by 2) operation. This is followed by the classification module, consisting of a fully connected layer of size 1024×200, ReLU activation, dropout, another fully connected layer of size 200×200, ReLU activation and a final fully connected layer of size 200×10. Effectively this network has 4 convolutional and 3 fully connected layers. We use batch size of 128 with this configuration.

For MNIST-ℓ_2 experiments, we consider the LeNet5 model from the Advertorch library (same as Ding et al. (2019)). This consists of a feature extractor of the form - two layers of 2-D convolutions, first one with 32 and second one with 64 channels, ReLU activation and maxpool by factor 2. The classifier consists of one fully connected layer of dimension 3136×1024 followed by ReLU activation, and finally another fully connected layer of size 1024×10. We use batch size of 50 with this configuration.

For CIFAR-ℓ_∞ experiments we consider a WideResNet with 34 layers and widening factor 10 (same as Zhang et al. (2019b) and Madry et al. (2018)). It consists of a 2-D convolutional operation, followed by 3 building blocks of WideResNet, ReLU, 2D average pooling and fully connected layer. Each building block of the WideResNet consists of 5 successive operations of batch normalization, ReLU, 2D convolution, another batch normalization, ReLU, dropout, a 2-D convolution and shortcut connection. We use batch size of 128 with this configuration.

For CIFAR-ℓ_2 experiments, we consider a ResNet with 20 layers. This ResNet consists of a 2-D convolution, followed by three blocks, each consisting of 3 basic blocks with 2 convolutional layers, batch normalization and ReLU. This is finally followed by average pooling and a fully connected layer. We use batch size of 256 with this configuration.

Training SGD and Entropy SGD models for MNIST experiments: For SGD, we trained the 7-layer convolutional network setup in Zhang et al. (2019b); Carlini and Wagner (2017) with the MNIST dataset,

setting batch size of 128, for ℓ_∞ SGD optimizer using a learning rate of 0.1, for 50 epochs. For Entropy SGD, with 5 langevin steps, and $\gamma = 10^{-3}$, batch size of 128 and learning rate of 0.1 and 50 total epochs.

A.2 ℓ_2 ATENT

ℓ_2-PGD attacks on CIFAR10: We explore the effectiveness of ℓ_2-ATENT as a defense against ℓ_2 perturbations. These results are tabulated in Table S1. We test 10-step PGD adversarial attacks at $\epsilon_2 = 0.5$ and $\epsilon_2 = 1$. For the purpose of this comparison, we compare pretrained models of MMA and PGD-AT at $\epsilon_2 = 1$. To train ATENT, we use $\gamma = 0.08$ for $\epsilon_2 = 1$, 10 step attack (with $2.5\epsilon_2/10$ step size), $K = 10$ langevin iterations, langevin step $\eta' = 2\epsilon_2/K$, learning rate for weights $\eta = 0.1$. ATENT achieves better robust accuracy against all baselines in Table S1. Specifically, the first three rows compare PGD, MMA and ℓ_2-ATENT against PGD attack of radii $\epsilon_2 = 0.5, 1$. All three models trained assume an attack budget of $\epsilon_2 = 1$. With this setting, all three models have similar performance for a weaker attack of $\epsilon_2 = 0.5$, whereas in the case of the stronger attack $\epsilon_2 = 1$, ATENT performs best. ATENT performs better even though we train a ResNet20 which has less expressive power as compared to WideResNet28. This shows that even with a smaller network, ATENT can produce a model that is more robust as compared to PGD-AT and MMA at high attack radii.

We also compare models primarily trained to boost the certificate of randomized smoothing. If the robust test accuracy is $\mathbf{1}_{y=f(x;w)}$ where $\mathbf{1}$ is an indicator vector with value 1 if $y = f(x;w)$ and 0 if $y \neq f(x;w)$, then the randomized smoothing *certified* robust accuracy is computed using a function $g(x) = \arg\max_{y \in \mathcal{C}} \mathbb{P}(f(x+\delta;w) - y)$, $\mathcal{C} = \{1, 2, \dots m\}$ possible labels, with $\delta \in \mathcal{N}(0, \sigma^2)$. In this case certified robust accuracy is $\mathbf{1}_{y=g(x;w)}$. TRADES (smoothing) (Blum et al. (2020)) algorithm aims to maximize this *certified* robust accuracy.

Even though ATENT algorithm by design does not maximize the certified robust accuracy, we test the generalization capablity of ATENT by comparing against smoothing version of TRADES (Blum et al. (2020)). For this we train a ResNet20 model for both TRADES smoothing version at default parameter setting and ℓ_2 ATENT, at $\eta' = 0.5\epsilon_2/K$, $\gamma = 0.05$, $\sigma(\varepsilon) = 0.577$ such that the effective noise standard deviation is 0.12. These models are tested against PGD-10 attacks at radius $\epsilon_2 = 0.435$. In all ℓ_2 ATENT experiments, we choose the value of $\gamma = 0.05$ such that the perturbation $\|X'^K - X\|_F \approx \epsilon_2$ of corresponding models of TRADES and PGD-AT. For all ATENT experiments, we set $\alpha = 0.9$. We see that ATENT does better in terms of standard robust accuracy.

Experiments on randomized smoothing: Since the formulation of ATENT is similar to a noisy PGD adversarial training algorithm, we test its efficiency towards randomized smoothing and producing a higher robustness certificate (Table S2). For this we train a ResNet-20 on CIFAR10, at $\gamma = 0.05, \eta' = 0.02, \eta = 0.1, K = 10$, and tune the noise ε, such that effective noise $\sqrt{2\eta'}\varepsilon$ has standard deviation $\sigma = 0.12$. We compare the results of randomized smoothing to established benchmarks on ResNet-110 (results have been borrowed from Table 1 of Blum et al. (2020)) as well as a smaller ResNet-20 model trained using TRADES at its default settings. We observe that without any modification to the current form of ATENT, our method is capable of producing a competitive certificate to state of art methods. Since ATENT does not solve the randomized smoothing objective, we cannot expect to see optimal *certified* robust accuracies; however we still see competitive performance. In future work we aim to design modifications to ATENT which can serve the objective of certification.

Table S2. Smoothed certified robust accuracies for CIFAR10 against ℓ_∞ attack of $\epsilon = 2/255$ ($\ell_2, \epsilon = 0.435$), smoothing factor $\sigma = 0.12$.

Smoothing radius → ↓ Defense	ResNet Type	Standard 0	ϵ_∞ 2/255
Crown IBP (Zhang et al. (2019a))	110	72.0	54.0
Smoothing (Wong et al. (2018))	110	68.3	53.9
SmoothAdv (Salman et al. (2019))	110	**82.1**	**60.8**
TRADES Smoothing (Blum et al. (2020))	110	**78.7**	**62.6**
TRADES Smoothing	20	**78.2**	**58.1**
ATENT (ours)	20	**72.2**	**55.41**

A.3 ℓ_∞ ATENT

Training characteristics of ℓ_∞ ATENT: In Figure S1 we display the training curves of ATENT. As shown, the robust accuracies spike sharply after the first learning rate decay, followed by an immediate decrease in robust accuracies. This behavior is similar to that observed in Rice et al. (2020). This is also the key intuition used in the design of the learning rate scheduler for TRADES.

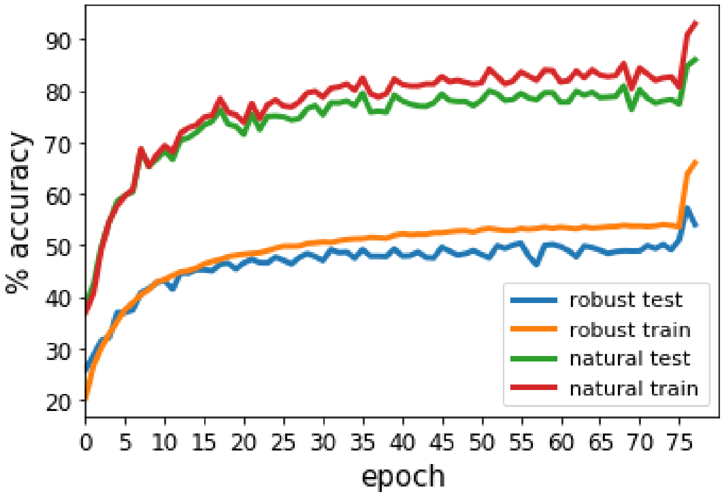

Figure S1: Benign training, test and robust training. test accuracies of ATENT. The learning rate is decayed at epoch 76, where the robust test accuracy peaks. This is the accuracy reported.

Table S3. Percentage robust accuracies for CIFAR10 against ℓ_∞ PGD and ATENT attacks of different radii.

Attack radius → ↓ Attack	2/255	4/255	8/255	12/255
PGD-20	79.83	73.35	57.23	39.37
ATENT-20	79.95	73.76	59.69	47.53

ATENT as Attack: For our ℓ_∞-ATENT WideResNet-34-10, we also test ℓ_∞-ATENT as an attack. We keep the same configuration as that of PGD-20, for ATENT. We compare the performance of our ℓ_∞-ATENT trained model (specifically designed to work against ϵ_∞=8/255 attacks). The values (Table S3) suggest that the adversarial perturbations generated by ATENT are similar in strength to those produced by PGD (worst possible attack).

Table S4. Average running time *per epoch* in seconds for ATENT against baselines for CIFAR10.

Algorithm	Total epochs	ℓ_2	ℓ_∞
ATENT(K=10)	76	282.04 ± 1.65	1128.21 ± 5.29
ATENT(K=5)	-	142.47 ± 1.18	572.85 ± 4.93
TRADES	100	2606.43 ± 2.64	1577.29 ± 1.45
PGD	150	241.79 ± 0.79	615.48 ± 4.09

Computational complexity: In terms of computational complexity, ATENT matches that of PGD and TRADES, as can be observed from the fact that all three approaches are nested iterative optimizations. In Table S4 we tabulate the running time performance of PGD (without random restarts), TRADES and attent, per epoch. We use default experiment settings for PGD and TRADES to make these comparisons. Note that because we rely on an early stopping criterion, there is no fair way of comparing overall running time of all baselines. ATENT requires only 76 epochs overall, whereas TRADES is run for 100 epochs and PGD is run for 150 epochs. The running time per epoch of ATENT is in between TRADES and PGD, making it competent even in terms of time-complexity. We also probe the running time dependence on choice of number of Langevin iterations K. We see that there is a linear dependence on K of the running time.

Due to the high computational complexity of all adversarial algorithms, we test a fine-tuning approach, to trade computational complexity for accuracy. This method is suggested in Jeddi et al. (2020). In this context, we take a pre-trained WideResNet-34-10 which has been trained on benign CIFAR10 samples only. This model is then fine tuned on adversarial training data, via ℓ_∞ ATENT using a low learning rate $\eta = 0.0001$ and trained for only 20 epochs. The final robust accuracy at $\epsilon_\infty = 8/255$ is 52.1%. This is accuracy marginally improves upon the robust accuracy observed (51.7%) for fine-tuned WideResNet-28-10 PGD-AT trained model in Jeddi et al. (2020). This experiments suggests that ATENT is amenable for fine tuning pretrained benign models using lesser computation, but at the cost of slightly reduced robust accuracy (roughly 5% drop at benchmark of $\epsilon_\infty = 8/255$).

B PROOFS AND DERIVATIONS

B.1 Theoretical properties of the augmented loss

We now state an informal theorem on the conditions required for convergence of SGLD in Eq. 7 for estimating adversarial samples X'. We restate Lemma 3.1 as follows:

LEMMA B.1. *The effective loss* $F(X'; X, Y, w) := \frac{\gamma}{2}\|X - X'\|_F^2 - \mathcal{L}(X'; Y, w)$ *which guides the Langevin sampling process in Eq. 7 is*

1. $\beta + \gamma$ *smooth if* $\mathcal{L}(X; Y, w)$ *is* β*-smooth in* X.
2. $\left(\frac{\gamma}{4}, \frac{L^2}{\gamma} + \frac{\gamma}{2}\|X\|_F^2\right)$ *dissipative if* $\mathcal{L}(X; Y, w)$ *is L-Lipschitz in* X.

One can then use smoothness and dissipativity of $F(X'; Y, w)$ to show convergence of SGLD for the optimization over X' (Eq. 7) via Theorem 3.3 of Xu et al. (2017).

We first derive smoothness conditions for the effective loss

$$F(X'; X, Y, w) := \frac{\gamma}{2}\|X - X'\|_F^2 - \mathcal{L}(X'; Y, w), \quad \forall X_1', X_2'.$$

We use abbreviations $p(X') := p(X'; X, Y, w), F(X') := F(X'; X, Y, w), \mathcal{L}(X'; Y, z) := \mathcal{L}(X')$ and $\mathcal{L}(X; Y, z) := \mathcal{L}(X)$, and assume that X and X' are vectorized. Unless specified otherwise, $\|\cdot\|$ refers to the vector 2-norm.

PROOF. Let us show that $\|\nabla_{X'} F(X'_2) - \nabla_{X'} F(X'_1)\| \leq \beta' \|X'_2 - X'_1\|$. If the original loss function is β smooth, i.e.,

$$\|\nabla_{X'} \mathcal{L}(X'_2) - \nabla_{X'} \mathcal{L}(X'_2)\| \leq \beta \|X'_2 - X'_1\|,$$

then:

$$\begin{aligned}
\|\nabla_{X'} F(X'_2) - \nabla_{X'} F(X'_1)\| &\leq \| - \nabla_{X'} \mathcal{L}(X'_2) + \nabla_{X'} \mathcal{L}(X'_1) - \gamma(X - X'_2) + \gamma(X - X'_1)\| \\
&\leq \|\nabla_{X'} \mathcal{L}(X'_2) - \nabla_{X'} \mathcal{L}(X'_1)\| + \|\gamma(X'_2 - X'_1)\| \\
&\leq (\beta + \gamma)\|X'_2 - X'_1\|
\end{aligned}$$

by application of the triangle inequality.

Next, we establish conditions required to show (m, b)-dissipativity for $F(X')$, i.e. $\langle \nabla_{X'} F(X'), X' \rangle \geq m\|X'\|_2^2 - b$ for positive constants $m, b > 0$, $\forall X'$. To show that:

$$\langle \nabla_{X'} F(X'), X' \rangle \geq m\|X'\|_2^2 - b$$

where the left side of inequality can be expanded as:

$$\begin{aligned}
\langle \nabla_{X'} F(X'), X' \rangle &= \langle -\nabla_{X'} \mathcal{L}(X') + \gamma(X' - X), X' \rangle \\
&= \langle -\nabla_{X'} \mathcal{L}(X'), X' \rangle + \gamma\|X'\|_2^2 - \gamma\langle X, X' \rangle \\
&= \langle -\nabla_{X'} \mathcal{L}(X'), X' \rangle + \gamma\|X'\|_2^2 - \frac{\gamma}{2}\left(\|X'\|_2^2 + \|X\|_2^2 - \|X - X'\|_2^2\right) \\
&\geq \langle -\nabla_{X'} \mathcal{L}(X'), X' \rangle + \gamma\|X'\|_2^2 - \frac{\gamma}{2}\left(\|X'\|_2^2 + \|X\|_2^2\right) \\
&= \langle -\nabla_{X'} \mathcal{L}(X'), X' \rangle + \frac{\gamma}{2}\|X'\|_2^2 - \frac{\gamma}{2}\|X\|_2^2
\end{aligned} \tag{S1}$$

To find the inner product $\langle -\nabla_{X'} \mathcal{L}(X'), X' \rangle$, we expand squares:

$$\begin{aligned}
&\|\nabla_{X'} \mathcal{L}(X') - \frac{\gamma}{2}X'\|^2 = \|\nabla_{X'} \mathcal{L}(X')\|_2^2 + \frac{\gamma^2}{4}\|X'\|_2^2 - \gamma\langle \nabla_{X'} \mathcal{L}(X'), X' \rangle \geq 0 \\
\implies &-\langle \nabla_{X'} \mathcal{L}(X'), X' \rangle \geq -\frac{\|\nabla_{X'} \mathcal{L}(X')\|_2^2}{\gamma} - \frac{\gamma}{4}\|X'\|_2^2
\end{aligned}$$

Plugging this into (S1), and assuming Lipschitz continuity of original loss $\mathcal{L}(X')$, i.e., $\|\nabla_{X'}\mathcal{L}(X')\|_2 \leq L$:

$$\begin{aligned}
\langle \nabla_{X'} F(X'), X' \rangle &\geq \langle -\nabla_{X'}\mathcal{L}(X'), X' \rangle + \frac{\gamma}{2}\|X'\|_2^2 - \frac{\gamma}{2}\|X\|_2^2 \\
&\geq -\frac{\|\nabla_{X'}\mathcal{L}(X')\|_2^2}{\gamma} - \frac{\gamma}{4}\|X'\|_2^2 + \frac{\gamma}{2}\|X'\|_2^2 - \frac{\gamma}{2}\|X\|_2^2 \\
&= \frac{\gamma}{4}\|X'\|_2^2 - \left(\frac{L^2}{\gamma} + \frac{\gamma}{2}\|X\|_2^2\right) \\
&= m\|X'\|_2^2 - b
\end{aligned}$$

where $m = \frac{\gamma}{4}$ and $b = \frac{L^2}{\gamma} + \frac{\gamma}{2}\|X\|_2^2$. Thus, $F(X')$ is $(\frac{\gamma}{4}, \frac{L^2}{\gamma} + \frac{\gamma}{2}\|X\|_2^2)$ dissipative, if $\mathcal{L}(X')$ is L-Lipschitz.

With Lemma B.1 we can show convergence of the SGLD inner optimization loop. To minimize overall loss function, the data entropy loss $\mathcal{L}_{DE}$ is minimized w.r.t. w, via Stochastic Gradient Descent (SGD). The gradient update for weights w are designed via Eq. 6 as follows:

$$\begin{aligned}
\nabla_w \mathcal{L}_{DE}(w; X, Y, \gamma) &= \nabla_w \int_{X'} \mathcal{L}(X'; Y, w) p(X'; X, Y, w, \gamma) dX' \\
&= \nabla_w \mathbb{E}_{X' \sim p(X'; X, Y, w, \gamma)}[\mathcal{L}(X'; Y, w)] \\
&= \int_{X'} \nabla_w \big(\mathcal{L}(X'; Y, w) p(X'; X, Y, w, \gamma)\big)\, dX' \\
&= \int_{X'} \nabla_w \mathcal{L}(X'; Y, w) \cdot p(X'; X, Y, w, \gamma) \\
&\quad + \nabla_w \mathcal{L}(X'; Y, w) \cdot \mathcal{L}(X'; Y, w) \cdot p(X'; X, Y, w, \gamma) dX' \\
&= \int_{X'} \nabla_w \mathcal{L}(X'; Y, w) \big(\mathcal{L}(X'; Y, w) + 1\big)\, p(X'; X, Y, w, \gamma) dX' \\
&= \mathbb{E}_{X' \sim p(X'; X, Y, w, \gamma)} \big(\nabla_w \mathcal{L}(X'; Y, w) \cdot (\mathcal{L}(X'; Y, w) + 1)\big)
\end{aligned}$$

Then a loose upper bound on Lipschitz continuity of $\mathcal{L}_{DE}$ is $\|\nabla_w \mathcal{L}_{DE}(w; X, Y, \gamma)\|_2 \leq \bar{L}(R + 1)$, if original loss is $\bar{L}$-Lipschitz in w and $\mathcal{L}(X) \leq R$. Due to the complicated form of this expression, establishing β-smoothness will require extra rigor. We push a more thorough evaluation of the convergence of the outer SGD loop to future work.

B.2 Entropy SGD

Chaudhari et al. (2019) claim that neural networks that favor wide local minima have better generalization properties, in terms of perturbations to data, weights as well as activations. Mathematically, the formulation in Entropy SGD can be summarized as follows. A basic way to model the distribution of the weights of the neural network is using a Gibbs distribution of the form:

$$p(w; X, Y, \beta) = Z_{X,\beta}^{-1} \exp\big(-\beta \mathcal{L}(w; X, Y)\big)$$

Algorithm 1 Entropy SGD

Input: $X = [X_{B_1}, X_{B_2} \dots X_{B_J}], f, \eta, \eta', w = w^0, \gamma, \alpha, \varepsilon$
for $t = 0, \cdots T-1$ **do**
  for $j = 1, \cdots J$ **do**
    $w'^0 \leftarrow w^t, \mu^0 \leftarrow w^t$ {Repeat inner loop for all training batches j}
    for $k = 0, \cdots, K-1$ **do**
      $dw'^k \leftarrow \frac{1}{n_j} \sum_{i=1}^{n_j} -\nabla_{w=w^k} L(f(w; x_i)) + \gamma(w^k - w'^k)$ $\{\forall x_i \in X_{B_j}\}$
      $w'^{k+1} \leftarrow w'^k + \eta' dw'^k + \sqrt{2\eta'}\varepsilon \mathcal{N}(0,1)$ {Langevin update}
      $\mu^k \leftarrow (1-\alpha)\mu^k + \alpha w'^{k+1}$
    end for
    $\mu^t \leftarrow \mu^K$
    $w^{t+1} \leftarrow w^t - \eta\gamma(w^t - \mu^t)$ {Repeat outer loop step for all training batches j}
  end for
end for
Output $\hat{w} \leftarrow w^T$

When $\beta \rightarrow \infty$, this distribution concentrates at the global (if unique) minimizer of $\mathcal{L}(w^*; X, Y)$. A modified Gibbs distribution, with an additional smoothing parameter is introduced, which assumes the form:

$$p(w'; w, X, Y, \beta = 1, \gamma) = Z^{-1}_{w,X,\gamma} \exp\left(-\mathcal{L}(w'; X, Y) - \frac{\gamma}{2}\|w' - w\|_2^2\right) \tag{S2}$$

where $Z_{w,X,\gamma}$ normalizes the probability.

Here γ controls the width of the valley; if $\gamma \rightarrow \infty$, the sampling is sharp, and this corresponds to no smoothing effect, meanwhile $\gamma \rightarrow 0$ corresponds to a uniform contribution from all points in the loss manifold. The standard objective is:

$$\begin{aligned} \min_w \mathcal{L}(w; X, Y) &:= \min_w - \log\Big(\exp\big(-\mathcal{L}(w; X, Y)\big)\Big) \\ &= \min_w - \log\left(\int_{w'} \exp\big(-\mathcal{L}(w'; X, Y)\big)\delta(w - w')dw'\right) \end{aligned}$$

which can be seen as a sharp sampling of the loss function. Now, if one defined the Local Entropy as:

$$\begin{aligned} \mathcal{L}_{ent}(w; X, Y) &= -\log(Z_{w,X,Y,\gamma}) \\ &= -\log\left(\int_{w'} \exp\left(-\mathcal{L}(w'; X, Y) - \frac{\gamma}{2}\|w - w'\|_2^2\right)dw'\right) \end{aligned}$$

our new objective is to minimize this augmented objective function $\mathcal{L}_{ent}(w; X, Y)$, which resembles a smoothed version of the loss function with a Gaussian kernel. The SGD update can be designed as follows:

$$\begin{aligned}\nabla_w \mathcal{L}_{ent}(w; X, Y) &= -\nabla_w(\log(Z_{w,X,Y,\gamma})) \\ &= Z^{-1}_{w,X,\gamma} \nabla_w(Z_{w,X,\gamma}) \\ &= Z^{-1}_{w,X,\gamma} \left(\int_{w'} e^{\left(-\mathcal{L}(w';X,Y) - \frac{\gamma}{2}\|w-w'\|_2^2\right)} \cdot \gamma(w - w')dw' \right) \\ &= \int_{w'} p(w'; w, X, Y, \gamma) \cdot \gamma(w - w')dw' \\ &= \mathbb{E}_{w' \sim p(w')} \left[\gamma(w - w')\right]\end{aligned}$$

Then, using this gradient, the SGD update for a given batch is designed as:

$$w^+ = w - \eta \nabla_w \mathcal{L}_{ent}(w; X, Y)$$

This gradient ideally requires computation over the entire training set at once; however can be extended to a batch-wise update rule by borrowing key findings from Welling and Teh (2011). This expectation for the full gradient is computationally intractable, however, Euler discretization of Langevin Stochastic Differential Equation, it can be approximated fairly well as

$$w'^{t+1} = w'^{t} + \eta^t \nabla_{w'} \log p(w'^t) + \sqrt{2\eta}\mathcal{N}(0, \mathbb{I})$$

such that after large enough amount of iterations $w^+ \to w^\infty$ then $w^\infty \sim p(w')$. One can estimate $\mathbb{E}_{w' \sim p(w')} \left[\gamma(w - w')\right]$ by averaging over many such iterates from this process. This result is stated as it is from (Chaudhari et al. (2019)): $\mathbb{E}_{w' \sim p(w')}[g(w')] = \frac{\sum_t \eta_t g(w'_t)}{\sum_t \eta_t}$. This leads to the algorithm shown in Algorithm 1. One can further accrue exponentially decaying weighted averaging of $g(w'_t)$ to estimate $\mathbb{E}_{w' \sim p(w')}[g(w')]$. This entire procedure is described in Algorithm 1.

This algorithm is then further guaranteed to find wide minima neighborhoods of w by design, as sketched out by the proofs in Chaudhari et al. (2019).

B.3 Stochastic Gradient Langevin Dynamics

Stochastic Gradient Langevin Dynamics combines techniques of Stochastic Gradient Descent and Langevin Dynamics (Welling and Teh (2011)). Given a probability distribution $\pi = p(\theta; X)$, the following update rule allows us to sample from the distribution π:

$$\theta^{t+1} = \theta^t + \eta \nabla_\theta p(\theta^t; X) + \sqrt{2\eta'}\varepsilon \tag{S3}$$

where η' is step size and ε is normally distributed. Then, as $t \to \infty$, $\theta \sim \pi$.

While this update rule in itself suffices, if the parameters are conditioned on a a training sample set X, which is typically large, the gradient term in Eq. S3 is expensive to compute. (Welling and Teh (2011)) shows that the following batch-wise update rule:

$$\theta^{t+1} = \theta^t + \eta \nabla_\theta p(\theta^t; X_{B_j}) + \sqrt{2\eta'}\varepsilon$$

Algorithm 2 PGD AT

1: **Input:** $[X_{B_1}, X_{B_2} \dots X_{B_J}], f, \eta, \eta', w = w^0, \epsilon$
2: **for** $t = 0, \cdots T-1$ **do**
3:   **for** $j = 1, \cdots J$ **do**
4:     $x'^0 \leftarrow x \; \{\forall x \in X_{B_j}\}$
5:     **for** $k = 0, \cdots, K-1$ **do**
6:       $dx'^k \leftarrow \frac{1}{n}\sum_{i=1}^{n} \nabla_{x=x^k} L(f(w^t; x))$
7:       $x'^{k+1} \leftarrow x'^k + \eta' dx'^k$ {Gradient ascent}
8:       project$_{x+\Delta}(x', \epsilon)$
9:     **end for**
10:     $\mu^t \leftarrow L(w^t, x'^K)$ {batch loss for X_{B_j}}
11:     $dL^t \leftarrow \nabla_w \mu^t$ {gradient of batch loss}
12:     $w^{t+1} \leftarrow w^t - \eta dL^t$
13:   **end for**
14: **end for**
15: **Output** $\hat{w} \leftarrow w^T$

suffices to produce a good approximation to the samples $\theta^{t\to\infty} \sim \pi$. In Algorithm 1 of main paper (ATENT), θ is the set of perturbed points X'. In each internal iteration, we look at subset of trainable parameters X'_{B_j}. We update the estimate for X'_{B_j} by only considering data-points X_{B_j} at a time. In the current formulation the set of iterable parameters X'_{B_j} only 'see' a single batch of data X_{B_j} ; a better estimate would require X'_{B_j} to be updated by iteratively over all possible batches X_{B_k}, $k = 1, 2....J$. However in practice we observe that just using the corresponding batch $X_{B_{k=j}}$ suffices. In future work, we will explore the theoretical implications of this algorithmic design.

B.4 PGD-Adversarial Training

In Algorithm 2 we describe the PGD-AT algorithm. Madry et al. (2018) demonstrate that PGD based-attack is the best possible attack that can be given for a given network and dataset combination. Theoretically,

$$\bar{x}_{\text{worst}} = \arg\max_{\delta \in \Delta_p} L(f(\hat{w}; x+\delta), y) \tag{S4}$$

and if this maximization can be solved tractably, then a network trained with the following min-max formulation is said to be robust:

$$\min_w \max_{\delta \in \Delta_p} \mathcal{L}(f(\hat{w}; X+\delta, Y), Y) \tag{S5}$$

Furthermore they show that first order based gradient approaches, such as SGD, are sufficient to suitably optimize the inner maximization over the perturbed dataset. This can be obtained using the following gradient *ascent* update rule:

$$X' = X' + \eta' \nabla_{X' \in X+\delta} \mathcal{L}(f(w, X'+\delta; Y), Y)$$

(see also Step 7 of Algorithm 2). Note that when $\Delta_p = \Delta_2$, this projection rule represents a noise-less version of the update rule in ATENT.

Iterative Fast Gradient Sign (IFGS) method effectively captures a similar projection based approach which performs an update within an ℓ_∞ ball. This update is given by:

$$X' = X' + \eta' \text{sign}(\nabla_{X'} \mathcal{L}(f(w, X' + \delta; Y), Y))$$

Note that this update rule constructs an adversarial example within ℓ_∞-ball, during the training procedure. Meanwhile, given our proposed adversarial example sampling criterion in Assumption 2, our update rule is slightly different.

C CONCURRENT WORK

We highlight the more recent developments in adversarial machine learning literature which were implemented concurrently with our work.

Adversarial attacks: Croce and Hein (2020) present a parameter-free approach to designing an adversarial attack against various classes of defenses. Apart from ℓ_2 and ℓ_∞ attacks, various papers propose other attack budgets, which use sparsity or ℓ_0 (Fan et al. (2020)). More recent papers discuss adversarial robustness of a new breed of classifiers such as vision transformers in terms of ℓ_2, ℓ_∞ perturbations (Shao et al. (2021); Paul and Chen (2021)) as well as sparsity based perturbations (Joshi et al. (2021)). Rony et al. (2021) reformulate adversarial attacks as an augmented Lagrangian problem, and show high attack success rates against defenses such as TRADES and adversarial training.

Adversarial defenses: Jiang et al. (2020) leverage contrastive learning for pretraining classifiers, and observe that self-supervised pretraining improves robustness to adversarial attacks. Li et al. (2021) propose to embed high dimnesional images into a low dimensional subspace and furhter regularize embeddings to improve robustness.

REFERENCES

Blum, A., Dick, T., Manoj, N., and Zhang, H. (2020). Random smoothing might be unable to certify l∞ robustness for high-dimensional images. *Journal of Machine Learning Research* 21, 1–21

Carlini, N. and Wagner, D. (2017). Towards evaluating the robustness of neural networks. In *2017 IEEE Symposium on Security and Privacy* (IEEE), 39–57

Chaudhari, P., Choromanska, A., Soatto, S., LeCun, Y., Baldassi, C., Borgs, C., et al. (2019). Entropy-sgd: Biasing gradient descent into wide valleys. *Journal of Statistical Mechanics: Theory and Experiment* 2019, 124018

Croce, F. and Hein, M. (2020). Reliable evaluation of adversarial robustness with an ensemble of diverse parameter-free attacks. In *International conference on machine learning* (PMLR), 2206–2216

Ding, G., Sharma, Y., Lui, K., and Huang, R. (2019). Mma training: Direct input space margin maximization through adversarial training. In *International Conference on Learning Representations*

Fan, Y., Wu, B., Li, T., Zhang, Y., Li, M., Li, Z., et al. (2020). Sparse adversarial attack via perturbation factorization. In *Computer Vision–ECCV 2020: 16th European Conference, Glasgow, UK, August 23–28, 2020, Proceedings, Part XXII 16* (Springer), 35–50

Jeddi, A., Shafiee, M., and Wong, A. (2020). A simple fine-tuning is all you need: Towards robust deep learning via adversarial fine-tuning. *arXiv preprint arXiv:2012.13628*

Jiang, Z., Chen, T., Chen, T., and Wang, Z. (2020). Robust pre-training by adversarial contrastive learning. In *NeurIPS*

Joshi, A., Jagatap, G., and Hegde, C. (2021). Adversarial token attacks on vision transformers. *arXiv preprint arXiv:2110.04337*

Li, Y., Min, M. R., Lee, T., Yu, W., Kruus, E., Wang, W., et al. (2021). Towards robustness of deep neural networks via regularization. In *Proceedings of the IEEE/CVF International Conference on Computer Vision (ICCV)*. 7496–7505

Madry, A., Makelov, A., Schmidt, L., Tsipras, D., and Vladu, A. (2018). Towards deep learning models resistant to adversarial attacks. In *International Conference on Learning Representations*

Paul, S. and Chen, P.-Y. (2021). Vision transformers are robust learners. *arXiv preprint arXiv:2105.07581*

Rice, L., Wong, E., and Kolter, Z. (2020). Overfitting in adversarially robust deep learning. In *International Conference on Machine Learning* (PMLR), 8093–8104

Rony, J., Granger, E., Pedersoli, M., and Ben Ayed, I. (2021). Augmented lagrangian adversarial attacks. In *Proceedings of the IEEE/CVF International Conference on Computer Vision (ICCV)*. 7738–7747

Salman, H., Li, J., Razenshteyn, I., Zhang, P., Zhang, H., Bubeck, S., et al. (2019). Provably robust deep learning via adversarially trained smoothed classifiers. In *Advances in Neural Information Processing Systems*. 11289–11300

Shao, R., Shi, Z., Yi, J., Chen, P.-Y., and Hsieh, C.-J. (2021). On the adversarial robustness of visual transformers. *arXiv preprint arXiv:2103.15670*

Welling, M. and Teh, Y. (2011). Bayesian learning via stochastic gradient langevin dynamics. In *Proceedings of the 28th International Conference on Machine Learning (ICML)*. 681–688

Wong, E., Schmidt, F., Metzen, J., and Kolter, J. (2018). Scaling provable adversarial defenses. In *NeurIPS*

Xu, P., Chen, J., Zou, D., and Gu, Q. (2017). Global convergence of langevin dynamics based algorithms for nonconvex optimization. *arXiv preprint arXiv:1707.06618*

Zhang, H., Chen, H., Xiao, C., Gowal, S., Stanforth, R., Li, B., et al. (2019a). Towards stable and efficient training of verifiably robust neural networks. In *International Conference on Learning Representations*

Zhang, H., Yu, Y., Jiao, J., Xing, E., El Ghaoui, L., and Jordan, M. (2019b). Theoretically principled trade-off between robustness and accuracy. In *International Conference on Machine Learning*. 7472–7482
